# Muscle mass and function in patients with type 2 diabetes mellitus: a characteristic analysis based on sex, age, and BMI

**DOI:** 10.3389/fendo.2026.1888206

**Published:** 2026-07-22

**Authors:** Ning Lu, Bing-li Liu, Wen Zhang, Lan-lan Jiang, Jin-dan Wu

**Affiliations:** 1 Department of Endocrinology, Nanjing First Hospital, Nanjing Medical University, Nanjing, China; 2 Department of Endocrinology, Lishui District People’s Hospital of Nanjing, Nanjing, China

**Keywords:** age, body mass index, sarcopenia, sex, type 2 diabetes mellitus

## Abstract

**Objective:**

To investigate the characteristics and determinants of muscle mass and muscle function in patients with type 2 diabetes mellitus (T2DM), stratified by sex, age, and body mass index (BMI).

**Methods:**

This is a retrospective cross-sectional study included 838 T2DM patients treated at the Department of Endocrinology and Metabolism, Nanjing First Hospital. Data on body weight, grip strength, gait speed, and other relevant clinical parameters were collected. Regression analyses and subgroup comparisons were performed according to sex, age, and BMI categories to evaluate the features and associated factors of muscle mass and muscle function in T2DM patients.

**Results:**

The prevalence of sarcopenia was slightly higher in female T2DM patients than that in males. Binary logistic regression analysis showed that, in addition to advanced age and low BMI, decreased serum albumin (Alb), elevated alkaline phosphatase, and insulin use were also significant risk factors for sarcopenia in male patients with T2DM. Multivariate analysis showed that in males, decreased skeletal muscle index (SMI) was independently associated with older age, low Alb, low creatinine, and low BMI; in females, it was independently associated with older age, low triglycerides, and low BMI. Regarding handgrip strength, older age and higher HbA1c were associated factors in males, whereas older age and longer diabetes duration were associated factors in females. For gait speed, older age and low albumin were associated factors in males, whereas only older age was significant in females. Age-stratified analyses revealed that in males, SMI and handgrip strength remained stable between ages 40 and 60, then declined significantly after 60; in females, declines were observed from approximately age 50. Gait speed decreased after age 70 in both sexes. BMI-stratified analyses revealed that sarcopenia prevalence increases with lower BMI, particularly in males. In males, low handgrip strength and gait speed rose sharply at BMI <18.5; no such association was observed in females.

**Conclusion:**

Older age and low BMI are common risk factors associated with sarcopenia in T2DM patients. In males, monitoring of muscle mass and functional decline is warranted after age 60, particularly in the presence of low serum albumin and low BMI. Preventive awareness and measures should be initiated when females reach age 50.

## 1 Introduction

Type 2 diabetes mellitus (T2DM) is one of the most widespread chronic metabolic diseases worldwide, with its incidence increasing annually. It is estimated that by 2030, approximately 578 million people will have diabetes, and by 2045, this number will reach 700 million (1). As the population ages, the disease burden continues to grow. China currently has the largest number of people with diabetes, with 174 million projected by 2045 (2). While T2DM is commonly associated with overweight (1), complex pathophysiological mechanisms—including chronic inflammation, dysregulated muscle autophagy, oxidative stress, and apoptosis—predispose patients to sarcopenia (3). Sarcopenia is defined by decreased size and strength of skeletal muscle, as well as reduced physical capacity, and its prevalence among patients with T2DM ranges from 6.3% to 47.1%, with rates reaching up to 28% in individuals over 50 years old (4, 5). Previous studies have shown that T2DM-associated sarcopenia is closely associated with not only poor glycemic control, but also increased oxidative damage (6). According to the 2019 consensus of the Asian Working Group for Sarcopenia (AWGS) (7), the diagnosis and severity of sarcopenia are based on three indicators; muscle strength (e.g., grip strength), skeletal muscle mass index (SMI), and physical performance (e.g., gait speed). Compared with age-matched patients without diabetes, older patients with T2DM show markedly decreased skeletal muscle mass, diminished lower-body muscular force, impaired physical capability and slower gait speeds (8, 9). The loss of muscle mass and decline in muscle function significantly impair physical function and overall health status, and increase the risk of falls in older adults (10). Therefore, identifying the clinical characteristics and risk factors of low muscle mass among T2DM patients through stratified analysis is clinically important for early intervention.

In this study, participants were stratified by age, sex, and BMI to investigate the characteristics and determinants of muscle mass and muscle function in T2DM patients. The findings aim to provide a theoretical basis for the early identification and targeted intervention of high-risk populations for sarcopenia.

## 2 Subjects and methods

### 2.1 Study population

**Figure 1:**
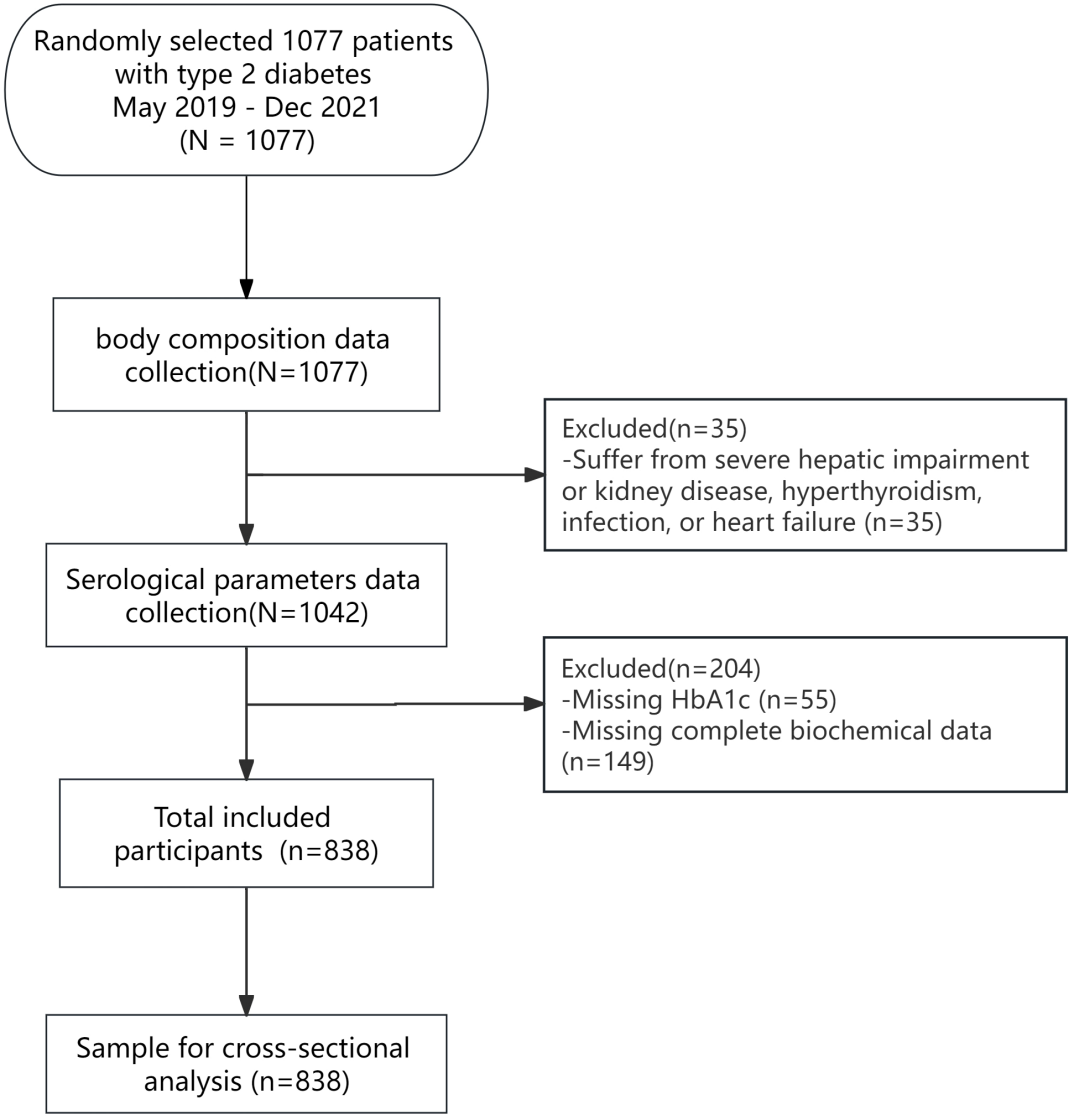
Flow diagram of the study participants.

A total of 1077 patients hospitalized in the Department of Endocrinology and Metabolism at Nanjing First Hospital between January 2019 and December 2021 underwent tests for sarcopenia. Of these, 35 patients were excluded because of severe hepatic impairment or kidney disease, hyperthyroidism, infection, or heart failure; 55 patients were excluded due to lack of data for HbA1c; 149 patients were excluded due to incomplete biochemical data; and 838 patients were finally enrolled for analysis(**Figure 1**).

Inclusion criteria were as follows:

1. Age ≥ 40 years;
2. The diagnosis of T2DM was established in accordance with the Chinese Guidelines for the Diagnosis and Treatment of Diabetes and was defined by any of the following criteria: typical symptoms of diabetes (polydipsia, polyuria, polyphagia, and unexplained weight loss) accompanied by a random blood glucose level ≥11.1 mmol/L; or fasting blood glucose (FBG) ≥7.0 mmol/L; or 2-hour plasma glucose ≥11.1 mmol/L during an oral glucose tolerance test (OGTT); or glycated hemoglobin (HbA1c) ≥6.5%;
3. Stable antihyperglycemic treatment regimen for at least 3 months;
4. Regular dietary habits and physical activity.

The exclusion criteria were as follows:

1. Acute diabetic complications, such as ketoacidosis and lactic acidosis;
2. Comorbidities affecting skeletal muscle metabolism, such as disuse muscle atrophy, severe cognitive disorder, and autoimmune disease;
3. Severe hepatic impairment or kidney disease, hyperthyroidism, infection, stress conditions or heart failure;
4. Malignant tumors;

Sarcopenia was diagnosed according to the AWGS (2019) criteria when there was low muscle mass (defined as skeletal muscle index < 7.0 kg/m2 in men and < 5.7 kg/m2 in women), together with either low muscle strength (defined as handgrip strength < 28 kg in men and < 18 kg in women) or low physical performance (defined as six-meter gait speed < 1.0 m/s) or both (7).

### 2.2 Research methods

#### 2.2.1 Collection of general information

Basic demographic and clinical data were recorded for each participant, including age, sex, height (m), body weight (kg), BMI, duration of disease (years), comorbidities, concomitant medications, and past medical history.

#### 2.2.2 Laboratory measurements

All blood samples were collected after at least 10 hours of overnight fasting. Blood biochemical analyses were performed in the hospital laboratory. The collected indicators included fasting blood glucose (FBG), triglycerides (TG), total cholesterol (TC), high-density lipoprotein cholesterol (HDL-C), low-density lipoprotein cholesterol (LDL-C), albumin (Alb), alkaline phosphatase (ALP), creatinine (Cr), hemoglobin (Hb) and HbA1c. HbA1c levels were determined using high-performance liquid chromatography (HPLC) (Bio-Rad Laboratories, Inc., CA, USA). C-peptide levels were measured by fluorescence immunoassay using the Modular Analytics E170 analyzer (Roche Diagnostics GmbH, Mannheim, Germany).

#### 2.2.3 Body composition and handgrip strength measurements

Body composition was assessed by trained professionals. Skeletal muscle mass (SMM) was measured using a multifrequency bioelectrical impedance analyzer (BIA; InBody 770, InBody Co., Ltd., Korea). The SMI refers to appendicular skeletal muscle mass index, and was calculated as SMM divided by height squared (kg/m²).

All BIA measurements were performed between 8:30 and 11:30 a.m, under standardized conditions (ambient temperature between 23–25 °C, fasting > 3 h, empty bladder, and supine position for at least 10 min) (11). None of the patients had edema, used diuretics, or received intravenous infusion within the past 3 months.

In addition, the following body composition parameters were also recorded: body fat mass (BFM), soft lean mass (SLM), fat-free mass (FFM), percent body fat (PBF), visceral fat area (VFA), fat mass index (FMI), lean mass index (LMI), and fat-free mass index (FFMI).

Handgrip strength was measured using a calibrated dynamometer. Participants were instructed to stand upright with the arm hanging naturally at the side of the body. Grip strength was measured three times for each hand, and the maximum value was recorded for analysis.

Gait speed was assessed using a 6-meter walk test conducted by trained research nurses. The time required to walk 6 meters was recorded with a stopwatch. The test was performed twice, and the average time was calculated. Gait speed (m/s) was determined by dividing the walking distance (6 m) by the average time required to complete the test.

### 2.3 Statistical analysis

Statistical analysis was done by using SPSS 26.0. The normality of continuous variables was assessed using the Shapiro - Wilk test. Normally distributed data are presented as mean ± standard deviation (SD), whereas non-normally distributed data are expressed as median (interquartile range, Q1–Q3). The Student t-test, Mann-Whitney U test or Chi-square test was used for comparisons between variables where appropriate. Categorical variables are presented as frequencies (percentages) and were compared using the chi-square (χ²) test. When the expected cell count was <5, the continuity-corrected chi-square test or Fisher’s exact test was applied, as appropriate. For continuous variables with normal distribution, Pearson correlation analysis was used to assess linear relationships between variables, whereas Spearman rank correlation analysis was applied for non-normally distributed variables. Candidate variables for regression models were selected based on clinical relevance, previous literature, and univariate screening (P < 0.10). Binary logistic regression was used to identify factors associated with sarcopenia, and multiple linear regression was applied for continuous outcome variables. Covariates were adjusted sequentially to assess confounding; a coefficient change > 10% was considered meaningful confounding. Multicollinearity was assessed using variance inflation factor (VIF < 10). Model fit for logistic regression was evaluated using the Hosmer–Lemeshow test and AUC. For linear models, residual normality, homoscedasticity, and influential outliers were checked using Q–Q plots, scatterplots of residuals, and Cook’s distance, respectively. Missing data were minimal (< 3%) and handled by complete−case analysis. Detection rates were described across subgroups. A two−sided P < 0.05 was considered statistically significant.

## 3 Results

### 3.1 General characteristics, comorbidities, and sarcopenia-related indicators

A total of 838 participants were included, comprising 477 men (56.9%) and 361 women (43.1%), with a mean age of 59 years. No significant differences were observed between sexes in age, duration of diabetes, HbA1c, TG, or FBG (all P>0.05). Compared with females, males had significantly higher levels of Alb, Hb, Cr, and FCP (all P<0.05). Conversely, females had elevated ALP, TC, LDL-C, and HDL-C (all P<0.05).

With respect to comorbidities, there were no significant differences between males and females in the prevalence of hypertension, fatty liver, coronary heart disease, diabetic nephropathy, and diabetic retinopathy (all P>0.05). However, females had a higher prevalence of cerebral infarction and diabetic peripheral neuropathy (both P<0.05), whereas males showed a higher prevalence of hyperlipidemia and peripheral arterial atherosclerosis (both P<0.05). Significant sex differences were observed in the utilization of sulfonylureas and acarbose (both P<0.05).

Regarding sarcopenia-related outcomes, the overall prevalence of sarcopenia was 19.69%, with a significantly higher prevalence in females than in males (23.27% vs. 16.98%, P = 0.023). The prevalence of low muscle mass did not differ significantly between sexes (P = 0.735), whereas females had significantly higher prevalence rates of low handgrip strength and low gait speed than males (P<0.001 and P = 0.004, respectively).

In terms of physical function and body composition, males exhibited significantly higher values of handgrip strength, gait speed, FFM, SMM, SMI, and FFMI (all P<0.05). On the contrary, females had larger values for BFM and PBF, as well as for VFA, with all differences between sexes being < 0.001. No significant difference in BMI was observed between sexes. Regarding glucose-lowering medications, the use of sulfonylureas and acarbose was significantly more prevalent in males than in females (P < 0.05). For the other antidiabetic agents, no significant sex−related differences were observed (P > 0.05)(**Table 1**).

**Table 1:** Characteristics of the participants according to sex.

Variable	Total	Male	Female	P value*
	(N = 838)	(N=477)	(N=361)	
Age (years)	59 (54,67)	58 (53,67)	61 (54.5,68)	0.054
Diabetes duration (years)	8 (2,12)	7 (2,12)	10 (2,13)	0.181
HbA1c(%)	8.8 (7.4,10.1)	8.7 (7.4,10.0)	8.8 (7.5,10.3)	0.075
Alb(g/L)	39.70 (37.80,41.90)	40.10 (38.00,42.53)	39.25 (37.50,41.23)	<0.001
Hb(g/L)	135 (125,145)	141 (131,149)	128 (120,136)	<0.001
ALP(U/L)	82 (66,99)	80 (65,96)	85 (70,105)	<0.001
TC(mmol/l)	4.38 (3.63,5.13)	4.26 (3.57,5.00)	4.58 (3.80,5.37)	<0.001
TG(mmol/l)	1.40 (0.97,2.14)	1.39 (0.92,2.16)	1.40 (1.01,2.11)	0.237
LDL-C(mmol/l)	1.95 (1.49,2.39)	1.91 (1.45,2.37)	1.99 (1.59,2.49)	0.024
HDL-C(mmol/l)	1.19 (1.01,1.39)	1.16 (0.96,1.33)	1.24 (1.05,1.48)	<0.001
Cr(umol/l)	64.20 (52.43,77.48)	70.70 (59.40,83.55)	54.50 (47.20,64.80)	<0.001
FBG(mmol/l)	8.01 (6.40,9.97)	7.91 (6.44,9.75)	8.20 (6.23,10.12)	0.410
FCP(ng/ml)	1.24 (0.82,1.82)	1.29 (0.85,1.87)	1.18 (0.76,1.78)	0.039
Comorbidities				
Hypertension (n,%)	442 (52.74)	248 (51.99)	194 (53.74)	0.616
Hyperlipidemia(n,%)	267 (31.86)	136 (28.51)	131 (36.29)	0.017
Fatty liver(n,%)	325 (38.78)	188 (39.41)	137 (37.95)	0.667
CVD(n,%)	125 (14.92)	73 (15.30)	52 (14.40)	0.717
Ischemic stroke(n,%)	164 (19.57)	78 (16.35)	86 (23.82)	0.007
DKD(n,%)	197 (23.51)	124 (26.00)	73 (20.22)	0.051
Retinopathy (n,%)	189 (23.05)	98 (21.08)	91 (25.63)	0.125
Neuropathy (n,%)	199 (23.75)	98 (20.55)	101 (27.98)	0.012
Atherosclerosis (n,%)	648 (77.33)	386 (80.92)	262 (72.58)	0.004
Muscle-related parameters				
Sarcopenia(%)	165 (19.69)	81 (16.98)	84 (23.27)	0.023
Low muscle mass(%)	211 (25.18)	118 (24.74)	93 (25.76)	0.735
Low Handgrip strength(%)	325 (38.78)	161 (33.75)	164 (45.43)	<0.001
Low Gait speed (%)	87 (10.38)	37 (7.76)	50 (13.85)	0.004
Handgrip strength (kg)	25.20 (18.88,32.80)	31.50 (26.35,36.10)	18.60 (15.85,22.55)	<0.001
Gait speed (m/s)	1.26 (1.14,1.37)	1.29 (1.18,1.40)	1.21 (1.10,1.33)	<0.001
BMI(kg/m2)	24.2 (22.1,26.5)	24.4 (22.5,26.4)	23.9 (21.7,26.6)	0.230
BFM(kg)	20.20 (16.10,25.60)	19.50 (15.70,24.55)	21.10 (17.00,26.60)	0.002
PBF(%)	29.85 (24.60,35.30)	26.80 (22.70,30.95)	34.90 (30.00,39.20)	<0.001
VFA(m2)	90.95 (69.38,121.93)	84.10 (67.00,107.70)	105.40 (75.20,138.90)	<0.001
FFM(kg)	45.15 (38.80,52.20)	50.90 (46.00,55.20)	38.80 (35.75,42.50)	<0.001
SMM(kg)	24.90 (21.18,28.83)	28.20 (25.45,30.85)	20.80 (19.05,22.80)	<0.001
SMI (kg/m2)	7.0 (6.3,7.7)	7.52 ± 0.74	6.28 ± 0.78	<0.001
FMI (kg/m2)	7.5 (5.9,9.5)	6.8 (5.5,8.5)	8.5 (6.8,10.6)	<0.001
FFMI (kg/m2)	16.86 ± 1.96	17.69 (16.43,18.86)	15.78 (14.82,16.85)	<0.001
Glucose−lowering drugs				
Insulin(n, %)	249 (29.71)	151 (31.66)	98 (27.15)	0.157
Metformin(n, %)	312 (37.23)	179 (37.53)	133 (36.84)	0.839
Sulfonylureas (n, %)	217 (25.89)	145 (30.40)	72 (19.94)	<0.001
Acarbose (n, %)	182 (21.72)	129 (27.04)	53 (14.68)	<0.001
TZD (n, %)	19 (2.27)	11 (2.31)	8 (2.22)	0.931
DPP-4i (n, %)	94 (11.22)	50 (10.48)	44 (12.19)	0.438
Glinides (n, %)	34 (4.06)	17 (3.56)	17 (4.71)	0.408
GLP-1 RA (n, %)	17 (2.03)	9 (01.89)	8 (2.22)	0.738
SGLT-2i (n, %)	34 (4.06)	16 (3.35)	18 (4.99)	0.236

HbA1c, hemoglobin A1c; Alb, Albumin; Hb, Hemoglobin;ALP, Alkaline phosphatase; TC, Total cholesterol; TG, Triglycerides; LDL-C, Low-density lipoprotein cholesterol; HDL-C, High-density lipoprotein cholesterol; Cr, creatinine; FBG, fasting blood glucose; FCP, fasting C-peptide; CVD, Cardiovascular Disease; DKD, Diabetic Kidney Disease; BMI, body mass index; BFM, Body Fat Mass; PBF,Percent Body Fat; VFA, Visceral Fat Area; FFM, Fat-Free Mass; SMM, Skeletal Muscle Mass; SMI, Skeletal Muscle Index; FMI, Fat Mass Index; FFMI, Fat-Free Mass Index; TZD, Thiazolidinediones; DPP-4i, Dipeptidyl peptidase-4 inhibitors; GLP-1 RA, Glucagon−like peptide-1 receptor agonists; SGLT-2i, Sodium-glucose cotransporter 2 inhibitors. Data are shown as the mean ± standard deviation or median (interquartile range) or percentage.

*male vs. female.

### 3.2 Multivariate linear regression analysis of factors associated with SMI, handgrip strength, and gait speed in men and women with T2DM

To account for potential confounding by glucose−lowering medications (including insulin, metformin, sulfonylureas, acarbose, DPP−4 inhibitors, GLP−1 receptor agonists, and SGLT−2 inhibitors) on the association between body composition parameters and sarcopenia, we performed chi−square tests stratified by sex and sarcopenia status. No significant differences in medication use were observed between the sarcopenia and non−sarcopenia groups for any of the agents (all P > 0.05).

Based on previous studies, sarcopenia is closely related to diabetes duration, blood glucose levels, diabetic complications, and nutritional status. Therefore, age, BMI, diabetes duration, HbA1c, diabetic complications, creatinine levels, albumin, and hypoglycemic drugs use were included in the logistic regression model as main confounding factors. Binary logistic regression analysis demonstrated that, in male patients, sarcopenia was significantly associated with advanced age, lower Alb levels, elevated ALP, and lower BMI (all P<0.05). Insulin use was associated with a reduced risk of sarcopenia in male T2DM patients. In female patients, sarcopenia was significantly associated with advanced age and lower BMI, while HbA1c and ALP showed no significant associations (all P>0.05) (**Table 2**).

**Table 2:** Binary logistic regression analysis of factors influencing sarcopenia.

	Variable	β	OR (95% CI)	P value
Total	Age (years)	0.097	1.102 (1.073,1.132)	<0.001
	Diabetes duration (years)	0.003	1.003 (0.972,1.035)	0.845
	HbA1c(%)	-0.032	0.969 (0.847,1.109)	0.646
	Alb(g/L)	-0.022	0.978 (0.916,1.044)	0.502
	ALP(U/L)	0.004	1.004 (0.996,1.011)	0.322
	Cr(umol/l)	-0.017	0.984 (0.972,0.995)	0.006
	FBG(mmol/l)	-0.041	0.96 (0.873,1.055)	0.393
	BMI(kg/m2)	-0.513	0.598 (0.541,0.662)	<0.001
	DKD	-0.294	0.745 (0.434,1.28)	0.287
	Retinopathy	0.187	1.205 (0.689,2.109)	0.513
	Neuropathy	0.041	1.042 (0.614,1.769)	0.878
	Insulin	-0.469	0.626 (0.383,1.021)	0.061
	Metformin	-0.036	0.965 (0.595,1.566)	0.885
	Sulfonylureas	0.296	1.345 (0.77,2.35)	0.298
	Acarbose	0.066	1.068 (0.6,1.9)	0.823
	TZD	-0.763	0.466 (0.14,1.55)	0.213
	DPP-4i	-0.264	0.768 (0.384,1.536)	0.455
	Glinides	-0.398	0.671 (0.213,2.114)	0.496
	GLP-1 RA	-0.256	0.774 (0.157,3.823)	0.754
	SGLT-2i	0.513	1.67 (0.459,6.071)	0.436
Male	Age (years)	0.128	1.137 (1.088,1.189)	<0.001
	Diabetes duration (years)	-0.046	0.955 (0.909,1.004)	0.071
	HbA1c(%)	-0.132	0.876 (0.724,1.06)	0.173
	Alb(g/L)	-0.126	0.882 (0.795,0.979)	0.018
	ALP(U/L)	0.011	1.011 (1,1.022)	0.047
	Cr(umol/l)	-0.014	0.987 (0.97,1.003)	0.108
	FBG(mmol/l)	0.044	1.045 (0.904,1.208)	0.551
	BMI(kg/m2)	-0.496	0.609 (0.521,0.712)	<0.001
	DKD	-0.733	0.481 (0.219,1.055)	0.068
	Retinopathy	0.104	1.11 (0.495,2.489)	0.801
	Neuropathy	-0.404	0.668 (0.309,1.444)	0.305
	Insulin	-0.819	0.441 (0.217,0.896)	0.024
	Metformin	0.064	1.066 (0.516,2.201)	0.863
	Sulfonylureas	0.369	1.447 (0.639,3.277)	0.376
	Acarbose	0.375	1.455 (0.629,3.368)	0.381
	TZD	-1.54	0.214 (0.038,1.225)	0.083
	DPP-4i	0.073	1.075 (0.355,3.254)	0.898
	Glinides	0.971	2.639 (0.262,26.615)	0.410
	GLP-1 RA	1.135	3.11 (0.074,131.175)	0.552
	SGLT-2i	18.98	174941236.395 (0,.)	0.998
Female	Age (years)	0.077	1.081 (1.039,1.123)	<0.001
	Diabetes duration (years)	0.031	1.031 (0.986,1.079)	0.182
	HbA1c(%)	0.051	1.053 (0.846,1.31)	0.645
	Alb(g/L)	0.08	1.083 (0.979,1.199)	0.122
	ALP(U/L)	0.001	1.001 (0.99,1.013)	0.832
	Cr(umol/l)	-0.019	0.981 (0.958,1.005)	0.127
	FBG(mmol/l)	-0.116	0.89 (0.772,1.026)	0.110
	BMI(kg/m2)	-0.519	0.595 (0.515,0.687)	<0.001
	DKD	0.086	1.089 (0.455,2.61)	0.848
	Retinopathy	0.17	1.186 (0.494,2.846)	0.703
	Neuropathy	0.421	1.524 (0.667,3.48)	0.317
	Insulin	0.015	1.015 (0.476,2.167)	0.969
	Metformin	-0.081	0.922 (0.44,1.931)	0.830
	Sulfonylureas	0.127	1.135 (0.484,2.661)	0.771
	Acarbose	-0.265	0.767 (0.299,1.965)	0.581
	TZD	-0.231	0.794 (0.109,5.763)	0.819
	DPP-4i	-0.461	0.631 (0.237,1.678)	0.356
	Glinides	-0.856	0.425 (0.099,1.822)	0.249
	GLP-1 RA	-0.845	0.43 (0.049,3.745)	0.444
	SGLT-2i	0.041	1.042 (0.24,4.514)	0.956

HbA1c, hemoglobin A1c; Alb, Albumin;ALP, Alkaline phosphatase; Cr, creatinine; FBG, fasting blood glucose; BMI, body mass index; DKD, Diabetic Kidney Disease; TZD, Thiazolidinediones; DPP-4i, Dipeptidyl peptidase-4 inhibitors; GLP-1 RA, Glucagon-like peptide-1 receptor agonists; SGLT-2i, Sodium-glucose cotransporter 2 inhibitors. OR, odds ratio; CI, confidence interval. P values were calculated using the Wald test. P < 0.05 was considered statistically significant.

Normality of continuous variables was assessed using the Shapiro–Wilk test. Pearson correlation analyses were applied to normally distributed variables, whereas Spearman rank correlation analyses were used for non-normally distributed variables to evaluate associations of SMI, handgrip strength, and gait speed with age, diabetes duration, BMI, and biochemical parameters. Based on these analyses, age, diabetes duration, HbA1c, Alb, ALP, TG, LDL-C, HDL-C, Cr, and BMI were included in multivariate linear regression models. Additionally, clinically important covariates such as diabetic complications and medication use were forced into the model to adjust for potential confounding, regardless of their statistical significance in univariate tests. Model 1 included the core metabolic and demographic variables, while Model 2 further adjusted for complications and antidiabetic agents. The final models (Model 2) are presented below.

Collinearity diagnostics showed variance inflation factors (VIF) all below 10 (range 1.04–1.47), indicating no substantial multicollinearity. Residual analyses using standardized residual plots and Q - Q plots suggested approximate normality and homoscedasticity. The Durbin–Watson statistics ranged from 1.763 to 2.049, indicating independence of residuals. Cook’s distances were <1, suggesting no influential outliers. These results support the validity of the linear regression assumptions.

For SMI in males, the model explained 62.5% of the variance (adjusted R²= 0.605, F(22,421) = 31.86, P < 0.001). Older age, lower Alb, lower Cr, and lower BMI were independently associated with decreased SMI, with SGLT2 inhibitor use showing borderline significance. In females, the model explained 67.8% of the variance (adjusted R²= 0.656, F(22,313) = 29.998, P < 0.001). Independent correlates of lower SMI included older age, higher ALP, lower TG, diabetic peripheral neuropathy, metformin use, and lower BMI. For handgrip strength in males, the model accounted for 21.4% of the variance (adjusted R²= 0.173, F(22,421) = 5.199, P < 0.001). Older age, lower Alb, and diabetic retinopathy were significantly associated with lower grip strength. In females, the model explained 24.9% of the variance (adjusted R²= 0.196, F(22,313) = 4.710, P < 0.001), with older age, longer diabetes duration, and lower BMI as independent determinants. For gait speed in males, the model explained 23.9% of the variance (adjusted R²= 0.199, F(22,420) = 5.996, P < 0.001). Older age, lower Alb, higher ALP, higher TG, and acarbose use were independently associated with slower gait speed. In females, the model explained 21.7% of the variance (adjusted R²= 0.162, F(22,313) = 3.943, P < 0.001); only older age remained significant, with no other variables reaching statistical significance (**Table 3**).

**Table 3:** Linear regression analysis of factors associated with muscle mass, grip strength, and gait speed in type 2 diabetes patients by gender.

Variables	Variable	R2	B	SE	Standardized β	95% CI	P
SMI (kg/m²)							
Male	Age (years)	0.605	-0.015	0.003	–0.199	-0.021 -(-0.010)	<0.001
	Alb (g/L)		0.013	0.007	0.067	0.000 - 0.026	0.050
	Cr (μmol/L)		0.003	0.001	0.097	0.001 - 0.006	0.003
	BMI (kg/m²)		0.163	0.008	0.698	0.148 - 0.178	<0.001
Female	Age (years)	0.656	-0.015	0.003	-0.198	-0.021 -(-0.010)	<0.001
	ALP (U/L)		-0.003	0.001	-0.123	-0.004 -(-0.001_	<0.001
	TG (mmol/L)		0.045	0.018	0.095	0.010 - 0.080	0.012
	Diabetic peripheral neuropathy		0.177	0.059	0.104	0.060 - 0.294	0.003
	Metformin		0.116	0.055	0.074	0.008 - 0.224	0.035
	BMI (kg/m²)		0.149	0.007	0.733	0.135 - 0.162	<0.001
Handgrip strength (kg)							
Male	Age (years)	0.173	-0.262	0.04	-0.342	-0.341 -(-0.183)	<0.001
	Alb (g/L)		0.233	0.096	0.119	0.044 - 0.421	0.016
	Diabetic retinopathy		-2.087	0.829	-0.116	-3.717 -(-0.458)	0.012
Female	Age (years)	0.196	-0.167	0.03	-0.306	-0.225 -(-0.108)	<0.001
	Diabetes duration (years)		-0.132	0.04	-0.185	-0.210 -(-0.054)	0.001
	BMI (kg/m²)		0.155	0.074	0.109	0.009 - 0.301	0.038
Gait speed (m/s)							
Male	Age (years)	0.199	-0.008	0.001	-0.361	–0.011 -(–0.006)	<0.001
	Alb (g/L)		0.013	0.003	0.222	0.007 - 0.018	<0.001
	TG (mmol/L)		-0.018	0.007	-0.121	-0.032 -(-0.003)	0.015
	ALP (U/L)		-0.001	0.000	-0.113	-0.002	0.013
	Acarbose		0.048	0.022	0.096	0.004 - 0.092	0.034
Female	Age (years)	0.162	-0.009	0.001	-0.393	-0.011 -(-0.006)	<0.001

SMI, skeletal muscle index; Alb, albumin; Cr, creatinine; BMI, body mass index; ALP, alkaline phosphatase; TG, triglycerides; CI, confidence interval. β, regression coefficient. P values were calculated using the t test. P < 0.05 was considered statistically significant.

### 3.3 Age- and sex-stratified analysis of muscle mass, handgrip strength, gait speed

Preliminary analyses of baseline characteristics showed no significant differences in age, BMI, or key covariates between sexes, suggesting that sex did not significantly interact with these variables in the multivariate models. Participants were stratified by sex and age to evaluate age−related changes in SMI, handgrip strength, and gait speed. They were categorized into four age groups: 40–49 years (69 men, 42 women), 50–59 years (193 men, 127 women), 60–69 years (129 men, 117 women), and ≥70 years (86 men, 75 women). Overall, with advancing age, the mean values of SMI, handgrip strength, and gait speed tended to decrease, while the proportions of participants with low SMI, low handgrip strength, and slow gait speed increased.

In males, SMI decreased slightly but not substantially from 40 to 59 years of age. From 40-49, it was 7.614 kg/m², declining to 7.174 kg/m² in those over 70. In females, however, SMI began to decline more rapidly around age 50. A similar pattern was observed for handgrip strength in men: from 40–59 it remained relatively stable at approximately 33.848 kg, and then declined markedly afterwards - from 33.848 kg down to just under 26.292 kg once they turn 60. In females, handgrip strength also goes down stepwise with age, starting at 29.628 kg in the 40–49 age group and reaching the lowest value of 21.496 kg in those over 70 years old. Concurrently, gait speed decreased steadily with age in both males and females, with a significant decline after age 70.

Age- and sex-stratified trends were also analyzed. In males, the percentage with low SMI remained relatively stable from age 40 to approximately 60, and increased gradually only after age 60 — until it exceeded that in women at or above age 70. In contrast, the prevalence of low SMI increased steadily with age in females. A similar pattern was observed for low handgrip strength.

**Figure 2:**
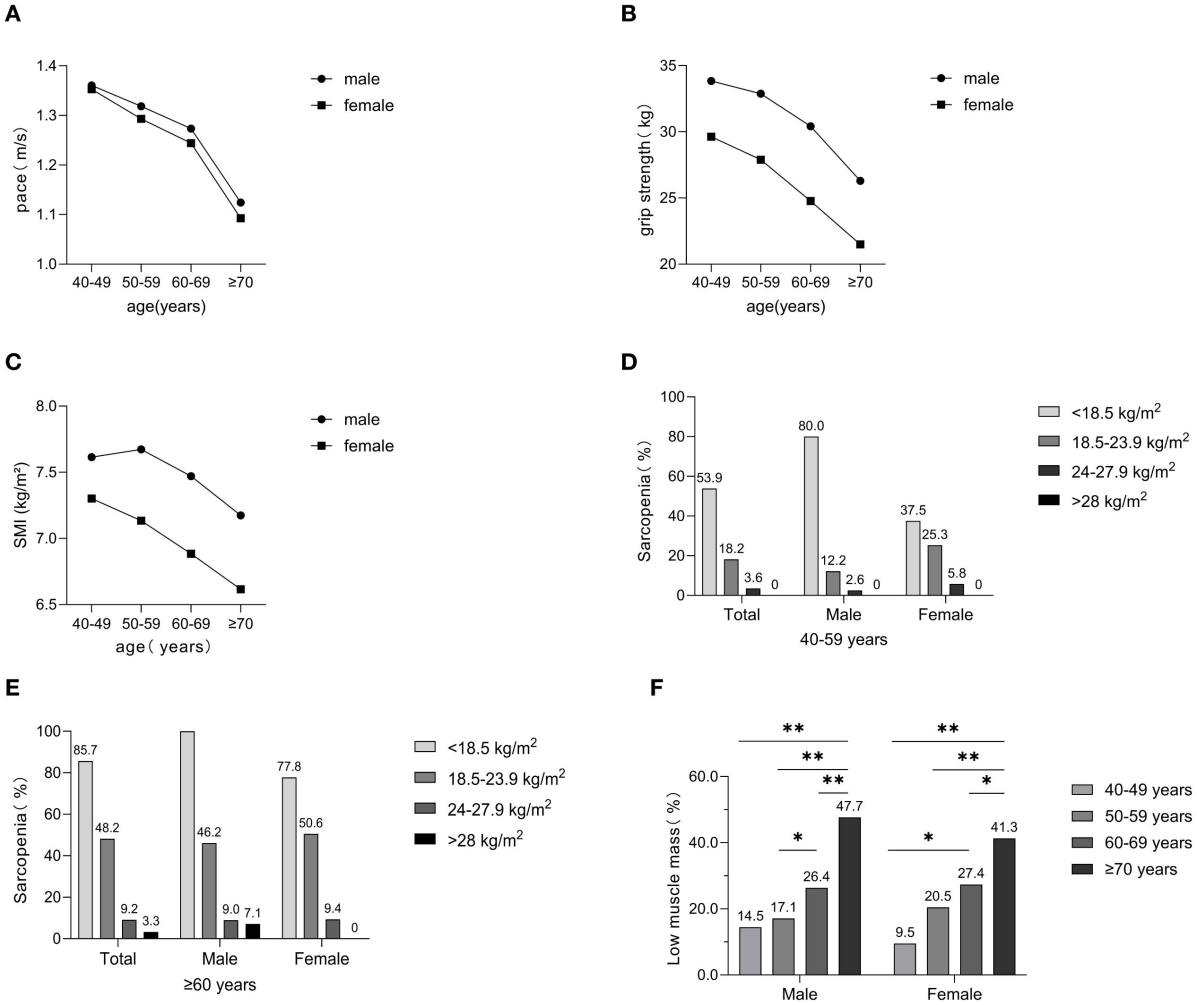
Age- and sex-related differences in skeletal muscle index, grip strength, and gait speed and the prevalence of their decline. **(A)** Skeletal muscle index (SMI) across different age groups in males and females. **(B)** Handgrip strength across different age groups in males and females. **(C)** Gait speed (pace) across different age groups in males and females. **(D)** Prevalence of low muscle mass among males and females in different age groups. **(E)** Prevalence of low handgrip strength among males and females in different age groups. **(F)** Prevalence of gait speed decline among males and females in different age groups. The mean value is calculated for continuous variables for statistical analysis. Data are presented according to four age groups: 40–49 years (69 men, 42 women), 50–59 years (193 men, 127 women), 60–69 years (129 men, 117 women), and ≥70 years (86 men, 75 women). Circles represent males and squares represent females in line graphs. Bar charts show the proportion of individuals with low muscle mass, low handgrip strength, or gait speed decline in each age group.*P < 0.05, **P < 0.01.

For gait speed, no participants in the 40–49 age group exhibited slow gait speed. However, in those over 70, the percentage increased to 20.93%. Females showed no notable increase until around age 70. Subsequently, the increase was more pronounced — from 4.76% to 41.33%, which exceeded the prevalence in males (**Figure 2**).

Sex−stratified correlation analyses were performed to examine the associations between fat−related parameters (BFM, PBF, VFA, and FMI) and sarcopenia−related indicators (SMI, grip strength, and gait speed). The results showed that, in both sexes, BFM, PBF, VFA, and FMI were all significantly and positively correlated with SMI (all P < 0.05). In males, PBF was significantly negatively correlated with both grip strength and gait speed (P < 0.05), and VFA was also significantly negatively correlated with gait speed (P < 0.05). In females, only BFM was significantly positively correlated with grip strength (P < 0.05), while gait speed showed no significant correlation with the four fat- related parameters (P > 0.05).

### 3.4 Sex- and BMI-stratified analysis of the prevalence of sarcopenia

We further looked at the number and distribution of sarcopenia cases across all ages, sex, and BMI categories. Participants were divided into four BMI groups: <18.5kg/m2 (10 men, 17 women), 18.5-23.9kg/m2 (202 men, 170 women), 24-27.9kg/m2 (195 men, 116 women), and ≥28kg/m2 (70 men, 58 women). In males, the prevalence of sarcopenia went up significantly as BMI went down among those aged 40-59. Also, in this age range, the rate in men was almost double what we saw in females of similar ages.

But what really popped out was the group at the very low end of the BMI scale - under 18.5 kg/m². About 80% of men in that category had sarcopenia, compared to only 37.5% of women. On the contrary, in females of the same age group, sarcopenia prevalence went up as BMI went down. But this was only seen until BMI fell below 24 kg/m². After that, there was a much larger increase.

**Figure 3:**
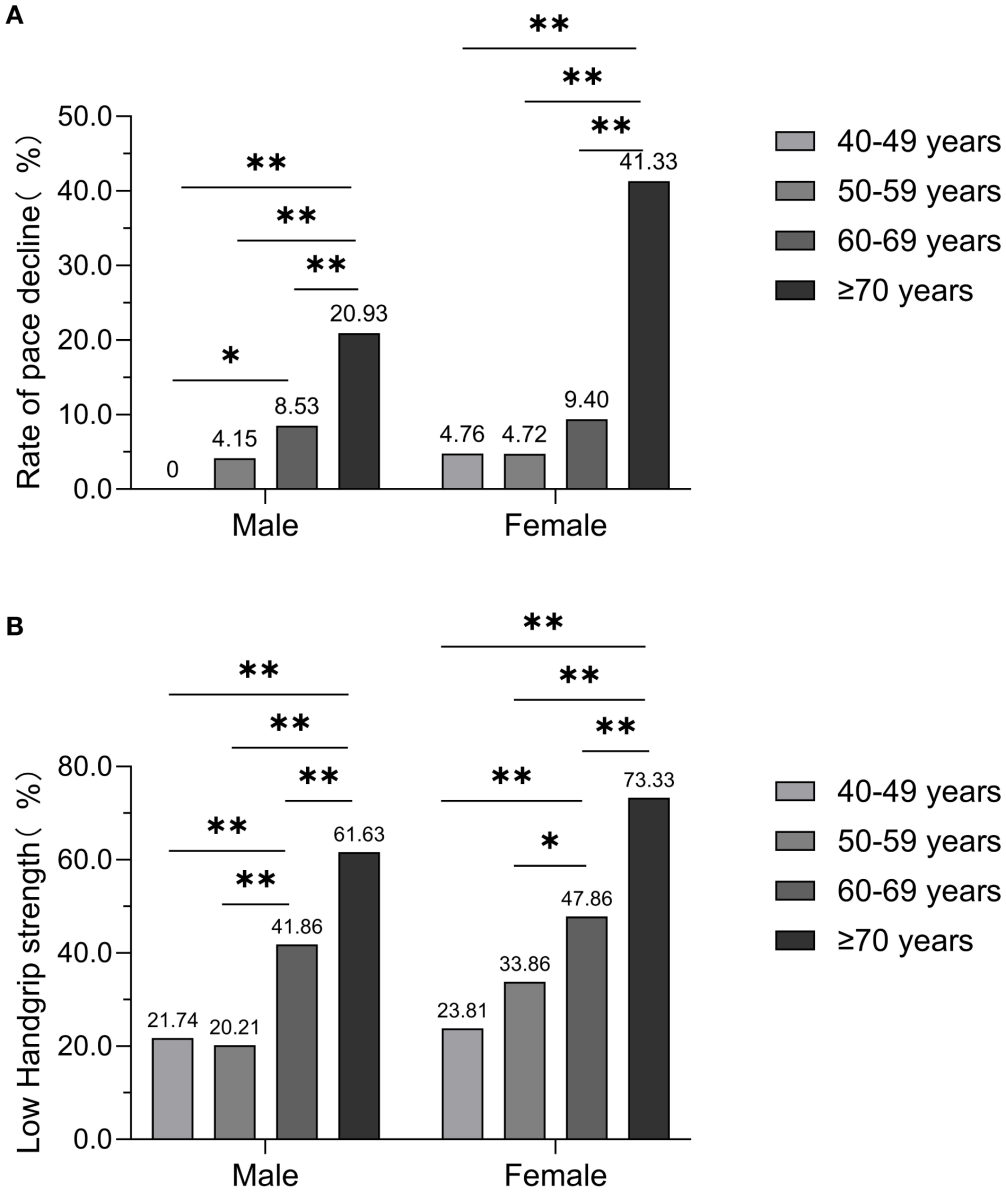
Prevalence of sarcopenia across different BMI categories by age and sex. **(A)** Prevalence of sarcopenia in individuals aged 40–59 years according to BMI categories in the total population, males, and females. **(B)** Prevalence of sarcopenia in individuals aged ≥60 years according to BMI categories in the total population, males, and females. BMI was categorized into four groups: <18.5kg/m2 (10 men, 17 women), 18.5-23.9kg/m2 (202 men, 170 women), 24-27.9kg/m2 (195 men, 116 women), and ≥28kg/m2 (70 men, 58 women). Bars represent the proportion of participants with sarcopenia in each BMI category. Comparisons were performed between BMI groups within each sex category. *P < 0.05, **P < 0.01.

In patients aged 60 and older, both men and women showed a steady increase in sarcopenia prevalence with lower BMI. Once again, the most noticeable jump occurred when BMI fell below 24 kg/m². (**Figure 3**)

### 3.5 Sex- and BMI-stratified analysis of the prevalence of low SMI, low handgrip strength, and low gait speed

Participants were further stratified by sex and BMI to examine the prevalence of low SMI, low handgrip strength, and low gait speed. Overall, BMI was strongly associated with all three sarcopenia components, with the highest prevalence consistently observed in individuals with BMI <18.5 kg/m² and a clear inverse relationship across increasing BMI categories.

Across both sexes, lower BMI was associated with a progressive increase in the prevalence of low SMI, particularly -- especially once BMI fell below 24 kg/m². In male patients, the relationship between BMI and handgrip strength was relatively stable when BMI was ≥18.5 kg/m²; however, the prevalence of low handgrip strength increased sharply when BMI fell below 18.5 kg/m². In contrast, in females, the prevalence of low handgrip strength increased with decreasing BMI, although the association did not reach statistical significance.

Regarding gait speed, BMI appeared to have little impact on male patients as long as it stayed at or above 23.9 kg/m², whereas the prevalence of low gait speed increased sharply when BMI fell below 18.5 kg/m². In female patients, the association between BMI and low gait speed was generally quite weak.

**Figure 4:**
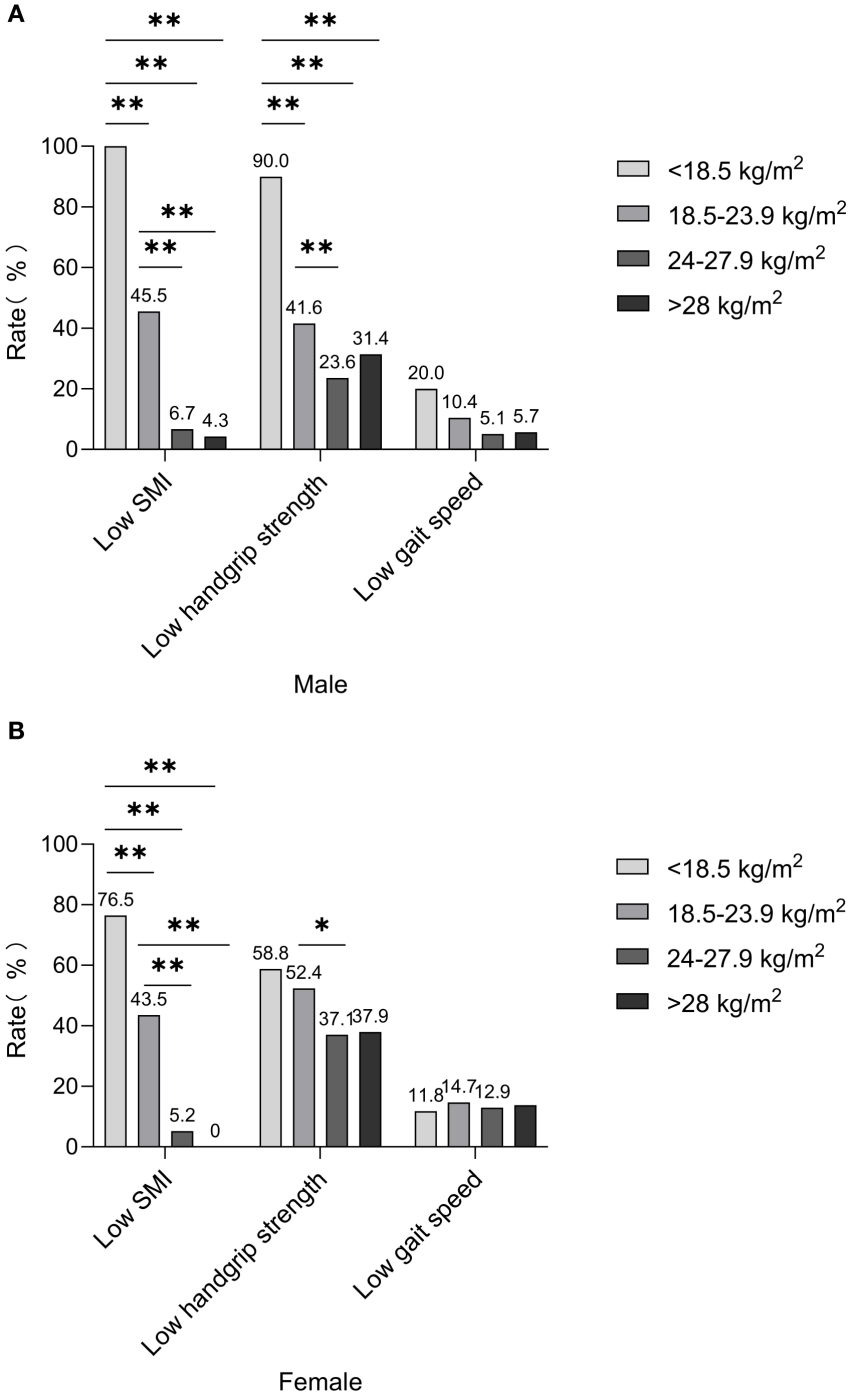
Prevalence of low skeletal muscle index, low handgrip strength, and low gait speed across different BMI categories by sex. **(A)** Prevalence of low SMI, low handgrip strength, and low gait speed in males across different BMI categories. **(B)** Prevalence of low SMI, low handgrip strength, and low gait speed in females across different BMI categories. BMI was categorized into four groups: <18.5kg/m2 (10 men, 17 women), 18.5-23.9kg/m2 (202 men, 170 women), 24-27.9kg/m2 (195 men, 116 women), and ≥28kg/m2 (70 men, 58 women). Bars represent the proportion (%) of participants with low SMI, low handgrip strength, and low gait speed within each BMI category.*P < 0.05, **P < 0.01.

In summary, BMI turned out to be a key factor influencing SMI, handgrip strength, and gait speed. Low BMI, particularly <18.5 kg/m², was associated with much higher prevalence of low SMI, low handgrip strength, and low gait speed. These effects were more pronounced in men, particularly when it came to low SMI (**Figure 4**).

## 4 Discussion

As the global population ages progressively, the incidence of diabetes is rising correspondingly. Sarcopenia, defined by an age-associated reduction in skeletal muscle mass, strength, and functional capacity (12), frequently co-occurs in middle-aged and elderly individuals diagnosed with T2DM. Current estimates indicate that sarcopenia affects approximately 10% to 16% of the elderly population worldwide, with a prevalence reaching approximately 18% among individuals with diabetes (13)The development of sarcopenia in individuals with T2DM is influenced by multiple factors. Previous research has identified key risk factors such as older age, male gender, longer duration of diabetes, diabetes-related complications like hypertension, and lack of physical activity, while a higher BMI might offer some protection (4, 14–16). Additionally, some studies have found that poor blood sugar control, indicated by increased HbA1c levels, is associated with muscle mass loss (17). The current body of evidence concerning sex differences in susceptibility to sarcopenia between males and females remains inconclusive. One study showed that sarcopenia was much more common in men (14, 18), whereas others reported it was more common in women (19, 20). Furthermore, several studies reported no statistically significant difference between sexes (21–25). These contradictory findings could be attributed to different study populations - like different ethnicities or ages.

Most prior studies on sarcopenia focused on the general population rather than differentiating by sex, thus limiting our understanding of sex-specific differences and associated risk factors. This study aims to investigate sex-specific differences in sarcopenia in a large cohort of hospitalized patients with T2DM.

Muscle mass and strength vary considerably between men and women due to various physiological factors. Testosterone and estradiol serve as key regulators of this process. constituting a basis for sex-based differences in muscle (26). In males, testosterone activates androgen receptors, leading to enhanced muscle protein synthesis and inhibition of protein degradation pathways. Concurrently, it stimulates satellite cell proliferation and differentiation, which contributes to muscle regeneration (27). After puberty, testosterone levels in males rise dramatically and continue to affect muscle size, protein synthesis rates, and strength gains throughout life. In females, estrogen is essential for muscle metabolism and mitochondrial function as well. Estrogen-mediated muscle repair begins at puberty and persists into old age. Estrogen supports muscle development and regeneration, maintains muscle homeostasis by reducing inflammation, maintaining mitochondrial stability, attenuating oxidative stress, and preserving satellite cell viability (28–30).

Furthermore, the effects of testosterone in men are associated with higher oxygen-carrying capacity, greater blood volume, and increased hemoglobin levels. These factors collectively support greater muscle mass and strength. However, females generally exhibit a higher percentage of body fat (31, 32), which may partially explain the sex differences in muscle mass and functional capacity.

This study examined 838 hospitalized patients with T2DM, all of whom underwent thorough initial assessments and evaluations for sarcopenia. The overall rate of sarcopenia was 19.69%. After adjusting for age, HbA1c levels, and BMI, female patients showed a significantly higher prevalence of sarcopenia compared to males(female, 23.27%; male, 16.98%, P = 0.023). Previous studies have demonstrated that ‘functional sarcopenia’ is more common in females, primarily manifested in elevated incidences of reduced grip strength and diminished gait speed (33, 34). Several factors may account for this observation. In particular, female patients with T2DM appear more vulnerable to intramuscular fat infiltration. Inflammation also inhibits muscle protein synthesis (35, 36). Moreover, reduced energy metabolic capacity, increased fatigue, and decreased stamina have been reported (35, 37).

In summary, these aforementioned mechanisms may explain why females experience a more marked reduction in muscle strength and a more rapid deterioration of lower limb muscle function compared to males (33, 38–40).

The findings of this study demonstrate that male patients with T2DM are not only more likely to be of advanced age and have lower BMI, but also more prone to exhibit decreased albumin levels and elevated ALP. This suggests that although nutritional and metabolic indicators are associated with sarcopenia prevalence in both sexes, their impact appears more pronounced in males. Given that inflammatory cytokines, bone turnover markers, vitamin D levels, and hepatobiliary disease status were not assessed in the present study, the following interpretations remain speculative. Previous studies confirmed that, elevated serum ALP is commonly indicative of hepatobiliary disorders or heightened bone metabolic activity. Additionally, ALP may have an indirect effect on the absorption of other nutrients such as lipids and proteins, through modulation of intestinal barrier integrity and gut microbiota composition. Several cross-sectional and cohort studies have corroborated the correlation between increased ALP levels and a higher prevalence of sarcopenia in the general population (41–43).

Based on our current findings in the T2DM cohort, high ALP may indicate a higher risk of sarcopenia. However, it should be emphasized, however, that ALP itself is unlikely to be a direct pathogenic factor. Rather, its elevation more probably reflects the cumulative effects of multiple underlying pathological processes—operating through various pathways—that collectively contribute to the development of sarcopenia.

The present study proposes the following mechanistic hypotheses. First, elevated ALP is commonly associated with systemic inflammation and metabolic stress (41). In patients with T2DM, chronic low-grade inflammation, oxidative stress, and insulin resistance act synergistically to enhance protein catabolism and suppress muscle protein synthesis (44). Thus, elevated ALP may serve as a surrogate marker of an imbalance favoring protein breakdown over anabolism, potentially exacerbating the progression of sarcopenia. Second, ALP is linked to arterial stiffness burden, and may impair muscle function via microvascular dysfunction, leading to reduced skeletal muscle perfusion and early fatigue during exercise. Indeed, previous studies have demonstrated a positive association between elevated ALP levels and an increased risk of arterial stiffness (45), which can compromise skeletal muscle blood flow and exercise tolerance, ultimately contributing to declines in muscle strength and mass.

In summary, these findings suggest that elevated ALP may serve as a clinical biomarker for increased risk of sarcopenia in male patients with T2DM.

Low Alb is a significant contributing factor to sarcopenia in male T2DM patients, and is widely used as an indicator of both nutritional status and inflammatory state (46, 47). Hypoalbuminemia is frequently identified as a risk factor for frailty (48). Epidemiological data indicate that the age-standardized incidence of protein-energy malnutrition is higher in Chinese males compared to females (49). Under conditions of excessive catabolism, the balance shifts toward protein breakdown rather than synthesis, disrupting skeletal muscle homeostasis and leading to atrophy.Furthermore chronic inflammation induces inflammatory cytokines, which subsequently activate the ubiquitin−proteasome pathway. Under these conditions, characterized by a negative acute-phase response, albumin levels decline rapidly, exacerbating muscle breakdown. Prior research has also shown a significant association between hypoalbuminemia and muscle weakness (46, 50). In patients with T2DM, insulin resistance impairs protein synthesis, thereby linking hypoalbuminemia to worsening sarcopenia.

Although albumin is not a direct etiological agent, it may function as a valuable predictive biomarker for sarcopenia in male individuals suffering from T2DM.

However, albumin concentrations are influenced by multiple factors, have a relatively prolonged half-life, and often exhibit delayed responsiveness to physiological changes. Moreover, most studies were cross-sectional in design, precluding causal inferences. Therefore, further longitudinal and mechanistic studies are warranted to elucidate these associations.

In males with T2DM, several factors have been identified as critical determinants of sarcopenia: older age, decreased Alb levels, lower BMI, and most notably, reduced Cr levels. Cr, which is synthesized from glycine, is predominantly distributed in muscle tissue. It is transported into muscle cells via glucose transporter type 4 (GLUT-4), facilitating glucose uptake and thereby enhancing muscle mass and function (51). Creatinine is the end-product of creatine metabolism (52). Previous studies have shown that patients with sarcopenia have significantly lower Cr levels than those without sarcopenia (53). This suggests that reduced Cr may serve as a clinical marker for sarcopenia in male T2DM patients. However, serum creatinine levels are not solely influenced by muscle mass; they also depend on renal function. Therefore, creatinine does not always accurately reflect muscle mass. In patients with renal insufficiency, impaired creatinine clearance results in higher serum Cr levels, which can mask the loss of muscle mass (54). Thus, renal function should be assessed concurrently when evaluating creatinine levels.

The results show that SGLT2 inhibitor use showed borderline significance (P = 0.050) for higher SMI in males, a finding that warrants further investigation given the growing interest in the pleiotropic effects of SGLT2 inhibitors on body composition. The positive association between metformin use and SMI in females is also notable; metformin may exert favorable effects on muscle metabolism via AMPK activation and anti−inflammatory properties (55), although further mechanistic studies are needed.

This study found that in men with T2DM, both SMI and handgrip strength - which reflect muscle mass and function - began to decline noticeably around age 60. In women, significant decreases in SMI and handgrip strength occurred earlier, at about 50 years old. This difference is likely attributable to sex-specific hormonal changes across the lifespan.

In men, androgen levels tend to decline gradually (56). Correspondingly, the gradual testosterone decline is associated with a decrease in skeletal muscle mass of approximately 0.5–1% per year after age 40, with an accelerated reduction after 60 (57). Additionally, changes in in myostatin concentration – a key regulator of muscle growth – contribute to the decline in muscle mass observed in males (58). Serum myostatin levels in males exhibit a modest increase with advancing age, followed by a decrease approximately at age 57, which may collectively contribute to the pronounced reduction in muscle mass and functional capacity in males older than 60 years of age. Muscle mass in women starts to decline at approximately age 30 (59), and estrogen levels decrease markedly at about 50 years of age. This is accompanied by an annual muscle mass loss of roughly 0.6% and a notable increase in abdominal fat (30, 60, 61). Estrogen is thought to protect against muscle mass loss and maintain muscle function, as well as regulate body fat distribution (62). Additionally, estrogen deficiency significantly impairs the maintenance, self-renewal, and differentiation of satellite cells, which ultimately leads to premature decline in the muscle mass and function (63). Therefore, given the marked decrease of estrogen levels observed in females at approximately 50 years of age, a corresponding pronounced decline in SMI, and grip strength occurs around this age. However, since the present study did not include data on menopausal status, age at menopause, hormone replacement therapy, history of oophorectomy, or circulating sex hormone levels, further large−scale real−world studies are warranted to confirm these findings.

Furthermore, stratification by BMI demonstrated that with decreasing BMI, SMI, handgrip strength, and gait speed declined to varying degrees in both men and women. However, when BMI was <18.5 kg/m², the prevalence of sarcopenia, low SMI, low handgrip strength, and low gait speed in male patients with T2DM was markedly higher than in female patients with T2DM. The prevalence of low SMI in male patients reached up to 100%, with a similarly high incidence of reduced grip strength of approximately 90% Moreover, the detection rate of sarcopenia among elderly males aged over 60 years was 100%. These results suggest the more pronounced negative effect of low BMI on muscle mass and function in males. The sex difference observed may be explained by complex interactions between biological factors, hormonal profiles, and lifestyle-related variables specific to male patients with T2DM. Women tend to have a substantially higher body fat percentage at any given BMI than men, who typically have more lean body mass. Since BMI does not distinguish between fat mass and lean mass, increases in BMI in men tend to more directly reflect changes in lean mass, whereas in women they more often reflect increases in fat mass (64). Consequently, both the absolute and relative reductions in muscle mass, alongside the increase in total adipose tissue, are more significant in males compared to females (19). A BMI below the normal threshold generally reflects inadequate energy intake or underlying metabolic dysfunctions. Malnutrition contributes not only to a reduction in muscle mass but also to an accelerated deterioration of muscle function (65). Given that male Chinese patients with T2DM are particularly vulnerable to protein-energy malnutrition (49), the incidence of sarcopenia among male T2DM patients appears to be more strongly influenced by nutritional factors.

In summary, the prevalence of sarcopenia in female patients with T2DM increases markedly after age 50 and is predominantly characterized by a decline in muscle function. In contrast, male patients with T2DM require greater attention to nutritional status, particularly adequate protein intake. These observations underscore the necessity of considering sex−specific characteristics in the prevention of sarcopenia among diabetic populations. Consequently, individualized nutritional and functional intervention strategies should be implemented to facilitate early detection and enable precision−based preventive and therapeutic approaches.

## 5 Study limitations

This study has several limitations. First, its cross-sectional design precludes the establishment of causal relationships between the identified factors and sarcopenia or declines in muscle function in patients with T2DM. Longitudinal studies are required to elucidate temporal associations and causality. Second, the study sample was drawn from a single center, and although stratified analyses by sex, age, and BMI were conducted, the relatively small sample sizes within certain subgroups may have limited statistical power and the generalizability of the findings. Third, the regression models did not adjust for several potential confounding factors, including physical activity, dietary intake, protein intake, nutritional assessment scores, inflammatory markers, vitamin D levels, renal function stage, diabetic neuropathy severity, cardiovascular disease severity, and other comorbidities, which may have influenced the observed associations with muscle mass and function. Finally, the estimation of the sarcopenia-related parameters relied primarily on the SMI, handgrip, gait speed which may not be the most sensitive measures, as they do not fully capture changes in muscle quality and neuromuscular function. Future research should incorporate broader evaluative methods and mechanistic biomarkers to better characterize sex-specific traits of sarcopenia in patients with T2DM.

## 6 Conclusion

In the present study population, the overall prevalence of sarcopenia was significantly higher in females than in males, and was predominantly characterized by impaired muscle function. Advanced age and lower BMI were commonly associated with sarcopenia in patients with T2DM. Specifically, in male patients older than 60 years, low serum albumin levels and reduced BMI were indicative of subsequent declines in muscle mass and function. In contrast, female patients exhibited muscle loss starting from age 50 onwards. Furthermore, compared with females, male T2DM patients presenting with hypoalbuminemia and elevated alkaline phosphatase (ALP) levels warranted increased vigilance regarding the risk of sarcopenia. Early detection and preventive strategies based on sex−specific muscle characteristics should be implemented to improve the quality of life in T2DM patients.

## Data Availability

The raw data supporting the conclusions of this article will be made available by the authors, without undue reservation.
